# Association of IGHG1 with carotid plaque progression and NK Cell-related immune features: Insights from bulk and single-cell transcriptomics

**DOI:** 10.1371/journal.pone.0353523

**Published:** 2026-08-03

**Authors:** Lun Shen, Yang Liu, Jingnan Xue, Luying Qi, Jiachen Wang, Yan Zhang, Wenli Sha, Yunfei Bao

**Affiliations:** Department of Radiology, First Affiliated Hospital of Huzhou Normal University, HuZhou, Zhejiang, China; Versiti Blood Research Institute, UNITED STATES OF AMERICA

## Abstract

**Background:**

The progression of carotid plaques is closely associated with immune dysregulation. This study aimed to identify key natural killer (NK) cell–related genes and to explore their immune microenvironment using bulk and single-cell transcriptomic data.

**Methods:**

Differential expression analysis was performed based on the transcriptomic dataset GSE43292. Key gene modules were identified using weighted gene co-expression network analysis (WGCNA). NK cell–related hub genes were further screened using Lasso and logistic regression, followed by validation in an external cohort. The expression profile of IGHG1 and intercellular communication of NK cells were assessed using single-cell RNA sequencing (scRNA-seq). Pseudotime trajectory analysis was conducted with Monocle 2, and IGHG1-associated signaling pathways were explored through gene set enrichment analysis (GSEA).

**Results:**

Four candidate genes were screened, and IGHG1 was the only independent risk factor. IGHG1 was significantly upregulated in advanced-stage plaques and demonstrated high predictive performance (AUC = 0.831; external AUC = 0.911). In the scRNA-seq dataset analyzed in this study, IGHG1 signal was mainly detected in the annotated NK-cell cluster. However, because IGHG1 is canonically associated with B-lineage and plasma cells, this observation should be interpreted cautiously in the context of SingleR/CellMarker-based annotation. Two NK-related subclusters were identified, showing inferred intercellular interactions predominantly involving the PPIA–BSG ligand–receptor pair. Pseudotime analysis revealed a transient high expression of IGHG1 during the transition from precursor to mature NK cells. GSEA indicated that IGHG1 was involved in the T cell receptor signaling pathway and NK cell–mediated cytotoxicity pathway.

**Conclusion:**

IGHG1 is a key gene closely associated with the progression of carotid plaques and was linked to NK cell-related immune features in our analyses. Given the canonical association of IGHG1 with B-lineage/plasma cells and the limitations of automatic annotation, its cellular origin and functional relevance require further validation.

## 1. Introduction

Carotid atherosclerosis (AS) is a progressive disease characterized by chronic inflammatory responses, primarily involving the accumulation of lipids within the arterial wall, leading to the formation of atherosclerotic plaques [1,2]. A typical atherosclerotic plaque consists of a necrotic core rich in extracellular lipids, covered by a fibrous cap composed of smooth muscle cells and collagen fibers [3]. As the disease progresses, inflammation-driven proteolytic enzyme activity increases, resulting in the degradation and thinning of the fibrous cap. This transition transforms plaques from a relatively stable state into a vulnerable or even ruptured condition [3,4]. Studies have demonstrated that advanced and unstable carotid plaques possess greater pathogenic potential than early-stage plaques and are associated with a significantly higher incidence of cerebrovascular events [5,6]. Epidemiological data indicate that approximately 18% to 25% of ischemic strokes are attributable to carotid atherosclerotic disease [7]. Early detection and intervention in plaque progression can effectively slow the development of AS and reduce the long-term burden of cerebrovascular diseases [8]. Therefore, elucidating the molecular mechanisms underlying carotid plaque progression and identifying predictive biomarkers is of great clinical significance.

In histopathological evaluations of vulnerable plaques, inflammation is considered a hallmark, characterized by the infiltration of immune cells such as monocytes, macrophages, and T lymphocytes [9]. Immune cell infiltration within the arterial wall is closely associated with plaque stability and the transition to high-risk, rupture-prone lesions [10]. Among various immune cells, natural killer (NK) cells—key components of the innate lymphoid system—are traditionally known for their ability to eliminate virus-infected and tumor cells. However, their role in the immunopathogenesis of AS has garnered increasing attention in recent years [11–13]. Previous studies have confirmed the presence of NK cells within atherosclerotic plaques [12,14]. Their expression of activating immune receptors contributes to local inflammatory responses, thereby promoting plaque development and progression [15,16]. Moreover, significant differences in NK cell infiltration and activation status have been observed between early and advanced carotid plaques, suggesting a potential role in modulating plaque stability [13]. Recent findings further indicate that NK cells may play a critical role in embolic strokes associated with carotid plaques [17]. Activated NK cells can secrete various pro-inflammatory cytokines (IFN-γ), which in turn polarize macrophages, amplify inflammation, and promote matrix metalloproteinases (MMPs) release, ultimately weakening the fibrous cap and facilitating plaque rupture [18,19]. Despite these advances, the precise molecular mechanisms through which NK cells contribute to plaque progression remain poorly understood.

In this study, we integrated bulk RNA sequencing and single-cell RNA sequencing (scRNA-seq) data to explore immune-associated transcriptional features related to carotid plaque progression, with a hypothesis-driven focus on NK cell-associated signals. By combining bulk transcriptomic screening, immune deconvolution, and single-cell contextualization, we aimed to identify candidate genes associated with plaque stage and to characterize their potential immune context in public datasets.

## 2. Methods

### 2.1. Dataset acquisition and preprocessing

The datasets used in this study were all obtained from the GEO database (https://www.ncbi.nlm.nih.gov/geo/). The transcriptomic data were derived from the GSE43292 dataset, which includes 64 human carotid atherosclerotic plaque samples—32 early-stage plaques (stage I or II) and 32 advanced-stage plaques (stage IV or above). Additionally, two external validation datasets were downloaded: GSE41571 (containing 5 ruptured plaques and 6 stable plaques) and GSE120521 (containing 4 unstable plaques and 4 stable plaques). Batch effects in the two external datasets were corrected using the removeBatchEffect function from the limma R package. The scRNA-seq data were obtained from the GSE159677 dataset, which includes calcified atherosclerotic core (AC) plaques and their matched proximal adjacent (PA) carotid tissues from 3 patients with carotid AS.

### 2.2. Differential expression analysis

Differentially expressed genes (DEGs) between early-stage and advanced-stage plaques were identified using the limma R package. The screening criteria were set as adjusted p < 0.05 and a fold change (FC) of 1.5. The ggplot2 package was used to visualize DEGs in a volcano plot.

### 2.3. Weighted gene co-expression network analysis (WGCNA)

To identify gene modules associated with carotid plaque progression, WGCNA was performed using the WGCNA R package. An appropriate soft-thresholding power was selected based on the scale-free topology criterion. A gene clustering tree was then constructed based on the topological overlap matrix, and genes were grouped into different modules using average linkage hierarchical clustering. Pearson correlation analysis was used to assess the relationship between each module and plaque stage, and modules significantly associated with disease progression were selected for further analysis.

### 2.4. Functional enrichment analysis

A Venn diagram was used to identify overlapping genes (carotid plaque-related genes) between the DEGs from early- and late-stage plaques and the genes from key WGCNA modules. Gene Ontology (GO) and Kyoto Encyclopedia of Genes and Genomes (KEGG) pathway enrichment analyses were performed using the clusterProfiler R package. p < 0.05 was set as the threshold for statistical significance. The enrichment results were visualized using bubble plots.

### 2.5. NK cell infiltration analysis and key gene identification

The CIBERSORT algorithm was applied to the normalized gene expression matrix to estimate immune cell infiltration. In this study, the terms “infiltration level” and “relative infiltration fraction” refer to the relative abundance estimates generated by CIBERSORT. Resting NK cells and activated NK cells were defined according to the preset immune cell categories in the LM22 reference signature matrix implemented in CIBERSORT. The Wilcoxon rank-sum test was used to compare the differences in NK cell infiltration between early- and late-stage plaques. To identify key genes associated with NK cells, activated NK cell infiltration levels were used as the dependent variable, and the carotid plaque-related DEGs were used as independent variables. Lasso regression analysis was conducted using the glmnet R package to select candidate genes. To avoid multicollinearity, the variance inflation factor (VIF) was calculated, and genes with VIF > 10 were excluded [20]. Univariate and multivariate logistic regression analyses were then performed on the selected genes to identify those independently associated with plaque progression. Genes with odds ratio (OR) > 1 were defined as risk-associated factors, whereas genes with OR < 1 were defined as protective-associated factors. The shapviz R package was used to calculate Shapley Additive exPlanations (SHAP) values to evaluate the importance of each gene.

### 2.6. Evaluation and validation of diagnostic performance

The expression differences of IGHG1 between early/late-stage plaques were analyzed. Receiver operating characteristic (ROC) curves and decision curve analysis (DCA) were used to assess the predictive performance and clinical utility of IGHG1. The diagnostic performance of IGHG1 was further validated in external datasets.

### 2.7. scRNA-seq analysis

The single-cell dataset GSE159677 was processed using the Seurat R package (v4.0). Quality control was performed based on the following criteria: 200 < nFeature_RNA < 5,000, 1,000 < nCount_RNA < 20,000, and percent.mt < 5%. The dataset was normalized using the NormalizeData function. Highly variable genes were identified using the FindVariableFeatures function with the “vst” method. Principal component analysis (PCA) was performed on the highly variable genes using the RunPCA function. The optimal number of principal components was determined using the JackStraw function (with p < 0.05). Cell clustering was conducted using the FindNeighbors and FindClusters functions, and t-distributed stochastic neighbor embedding (t-SNE) was used for visualization. Cell-type classification and annotation were performed using an automatic matching strategy based on SingleR and the CellMarker database. Briefly, after cell clustering, the expression profiles of single-cell clusters were compared with reference cell-type information derived from CellMarker, and cell labels were assigned according to the best-matched reference annotations. The expression of IGHG1 across different cell types and NK cell subsets was visualized using bubble plots. The expression of IGHG1 was also compared between PA and AC tissues.

### 2.8. Cell–cell communication and pseudotime analysis

Cell–cell communication differences between NK cell subsets were inferred using CellChat (version 2.1.0). Overexpressed ligands, receptors, and ligand–receptor interactions were identified using the identifyOverExpressedGenes and identifyOverExpressedInteractions functions. Pseudotime analysis was performed using Monocle 2 (version 2.28.0).

### 2.9. Gene set enrichment analysis (GSEA)

To further explore the potential biological functions and regulatory pathways of IGHG1, GSEA was performed based on KEGG pathways. Genes were ranked according to their correlation with IGHG1 expression, and KEGG gene sets were used for enrichment analysis with the clusterProfiler R package. p < 0.05 was considered statistically significant.

### 2.10. Statistical analysis

All statistical analyses were conducted using R software (version 4.2.3). A two-tailed p-value < 0.05 was considered statistically significant.

## 3. Results

### 3.1. Identification of DEGs associated with carotid plaque stages and their functional enrichment analysis

**Fig 1:**
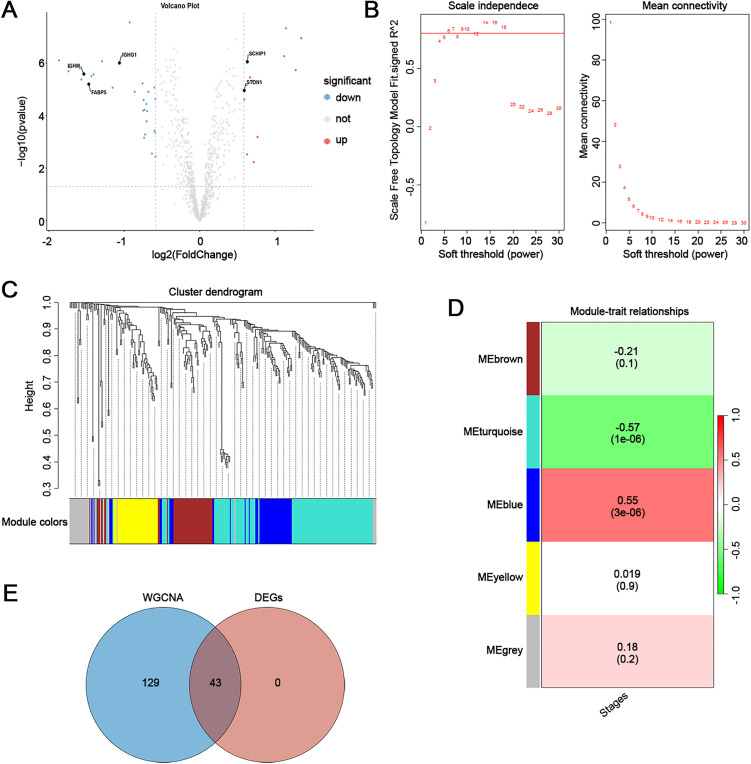
Identification of DEGs associated with carotid plaque stages. (A). Volcano plot shows the DEGs between the early-stage and advanced-stage of carotid plaque. (B). Selection of the soft-thresholding power in WGCNA. The left panel shows the scale-free topology fit index (R²) as a function of soft-threshold power. The right panel shows mean connectivity. (C). Gene dendrogram obtained by hierarchical clustering, with modules assigned by dynamic tree cut and shown in different colors. (D). Module-trait relationship heatmap. Correlation coefficients and p-values (in parentheses) between module eigengenes and disease stage are shown. E. Venn diagram showing the overlap between DEGs and genes in WGCNA modules.

Firstly, differential expression analysis was performed on the transcriptomic data from the GSE43292 dataset. A total of 43 DEGs were identified, including 13 upregulated and 30 downregulated genes (Fig 1A). Subsequently, WGCNA was conducted to further identify co-expression gene modules closely associated with the progression of carotid plaques. An appropriate soft-thresholding power was selected based on the scale-free topology criterion (Fig 1B). Based on this, a gene clustering tree was constructed using the topological overlap matrix, and four distinct co-expression modules were identified (Fig 1C). The module–trait relationship heatmap revealed that the turquoise module was significantly negatively correlated with carotid plaque stage (r = –0.57, p < 0.001), while the blue module was significantly positively correlated (r = 0.55, p < 0.01) (Fig 1D). The results suggest that these two modules may be involved in regulating carotid plaque progression. Therefore, the turquoise and blue modules were selected for subsequent analysis, comprising a total of 172 genes. Next, the genes from these two key modules were intersected with the identified DEGs, resulting in 43 overlapping genes (Fig 1E). The specific names of these overlapping genes are listed in S1 Table.

**Fig 2:**
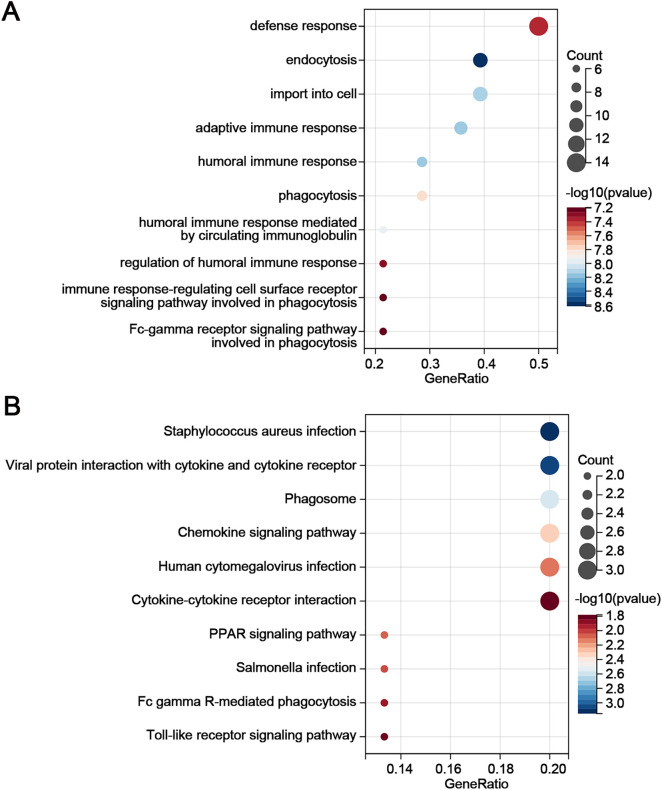
Functional enrichment analysis of carotid plaque-related DEGs. (A). Top 10 GO terms. (B). KEGG enrichment pathways.

To explore the potential functions and signaling pathways of the 43 overlapping genes, functional enrichment analysis was performed. GO analysis indicated that these genes were primarily enriched in biological processes such as defense response, endocytosis, import into cell, adaptive immune response, and humoral immune response (Fig 2A). KEGG analysis further revealed that these genes were significantly enriched in several immune-related pathways, including staphylococcus aureus infection, phagosome, chemokine signaling pathway, cytokine–cytokine receptor interaction, and Toll-like receptor signaling pathway (Fig 2B).

### 3.2. Identification of NK cell–related hub genes in carotid plaque

The functional enrichment results suggested that plaque stage-associated DEGs were mainly involved in immune-related pathways, indicating broad immune remodeling during carotid plaque progression rather than NK cell-specific transcriptional changes. To characterize this immune remodeling more comprehensively, we applied CIBERSORT to estimate the relative abundance of immune cell subsets in early-stage and advanced-stage plaques. The results showed that multiple immune cell populations exhibited significant differences between the two groups, including B-lineage cells, macrophages, dendritic cells, neutrophils, and NK cells (S1 Fig). These findings indicate that plaque progression is accompanied by complex changes in the immune microenvironment involving multiple immune cell types.

**Fig 3:**
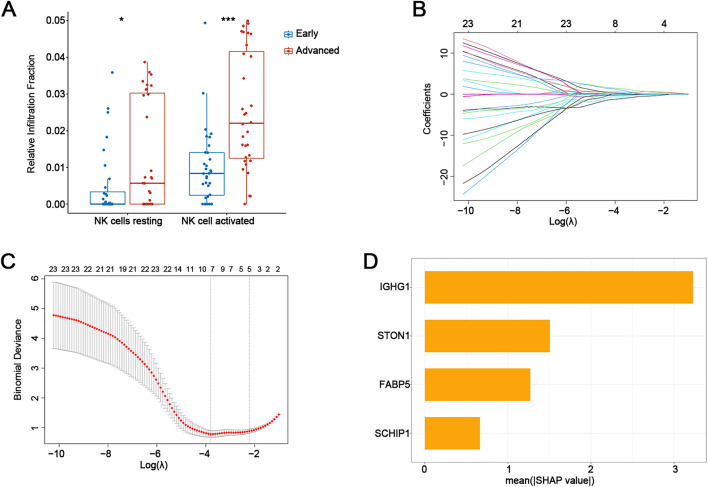
Identification of NK cell–related hub genes in carotid plaque. (A). Relative infiltration fraction of NK cells between early-stage and advanced-stage plaques; *p < 0.05, ***p < 0.001. (B-C). Lasso regression analysis. (D). SHAP analysis reveals the weight importance of candidate genes.

A previous study has reported that the presence of NK cells in human atherosclerotic plaques and their association with symptomatic or vulnerable carotid plaques. [13]. Consistent with this rationale, our CIBERSORT analysis showed that NK cells were also among the immune cell subsets altered during plaque progression (Fig 3A). Therefore, we further investigated NK-cell-associated transcriptional features by using the estimated activated NK-cell fraction as the response variable in the subsequent LASSO-based screening model. Subsequently, Lasso regression analysis was performed using the infiltration level of activated NK cells as the outcome variable, and included the previously identified 43 overlapping genes as input features (Fig 3B-C). This analysis identified five candidate genes: FABP5, IGHG1, STON1, SCHIP1, and IGHM. To further identify risk genes among these candidates, collinearity analysis was first conducted to avoid potential multicollinearity. The results showed strong collinearity between IGHG1 and IGHM, with VIF values of 13.120 and 13.248, respectively (Table 1). Therefore, IGHM was excluded, and collinearity analysis was repeated. The remaining four genes all had VIF values less than 10 (Table 1), indicating that collinearity among these genes was within an acceptable range. Next, univariate logistic regression analysis was performed for the remaining four genes (FABP5, IGHG1, STON1, and SCHIP1). All these genes were found to be significantly associated with carotid plaque staging (p < 0.001, Table 2). Among them, FABP5 and IGHG1 were identified as risk factors, while STON1 and SCHIP1 served as protective factors. Further multivariate logistic regression analysis revealed that IGHG1 was the only independent risk factor significantly associated with carotid plaque staging (p < 0.001, Table 2). SHAP analysis also demonstrated that IGHG1 had the highest feature importance among the four genes (Fig 3D). Therefore, subsequent analyses focused on IGHG1.

**Table 1:** VIF analysis before and after exclusion of IGHG1.

Model	Gene	VIF
Initial model	FABP5	4.529
Initial model	IGHG1	13.12
Initial model	STON1	2.733
Initial model	SCHIP1	4.727
Initial model	IGHM	13.248
Model excluding IGHG1	FABP5	3.495
Model excluding IGHG1	IGHM	1.644
Model excluding IGHG1	STON1	2.64
Model excluding IGHG1	SCHIP1	4.542

**Table 2:** Univariate and multivariate logistic regression analyses of candidate genes associated with carotid plaque stage.

Gene	Univariate OR	Univariate 95% CI	Univariate P value	Multivariate β	Multivariate OR	Multivariate 95% CI	Multivariate P value
FABP5	3.129	1.696–5.775	<0.001	0.577	1.78	0.415–7.628	0.437
IGHG1	16.211	4.647–56.546	<0.001	2.425	11.303	2.982–42.845	<0.001
STON1	0.078	0.021–0.298	<0.001	−0.668	0.513	0.050–5.269	0.574
SCHIP1	0.053	0.012–0.223	<0.001	0.008	1.008	0.038–26.541	0.996

### 3.3. IGHG1 is highly expressed in advanced carotid plaques and exhibits good predictive performance for plaque progression

**Fig 4:**
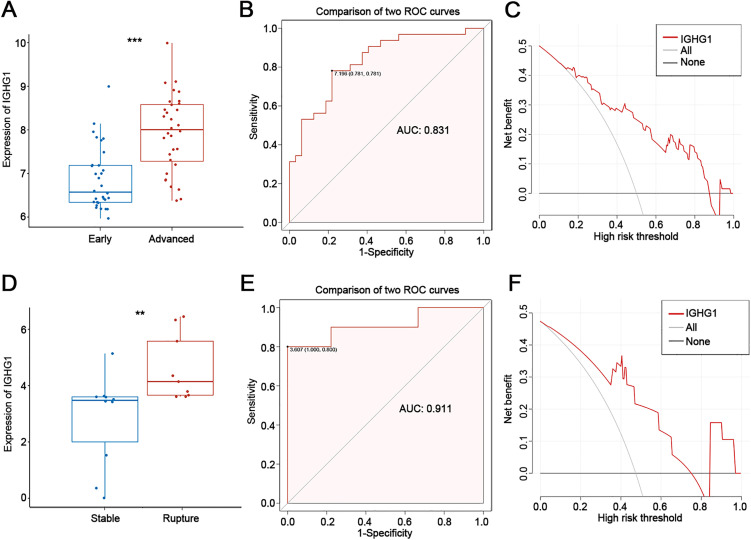
IGHG1 is highly expressed in advanced carotid plaques and exhibits good predictive performance for plaque progression. (A). Expression of IGHG1 between early- and advanced-stage carotid plaques; ***p < 0.001. (B). ROC curve reveals the predictive performance of IGHG1 on carotid plaque stages. (C). DCA curve reveals the net clinical benefit of IGHG1 expression across different risk thresholds. (D). Expression of IGHG1 between stable and rupture carotid plaques; **p < 0.01. (E). ROC curve reveals the predictive performance of IGHG1 on carotid plaque progression based on the external datasets. (F). DCA curve reveals the net clinical benefit of IGHG1 expression across different risk thresholds based on the external dataset.

To further investigate the potential role of IGHG1 in carotid plaque progression, we compared its expression levels between early- and advanced-stage plaques. As shown in Fig 4A, IGHG1 expression was significantly upregulated in advanced plaques compared to early-stage plaques (p < 0.001). Subsequently, ROC analysis was performed to assess the predictive performance of IGHG1 in distinguishing plaque stages. The results demonstrated good predictive ability, with an AUC of 0.831 (Fig 4B). DCA further confirmed the clinical utility of IGHG1, indicating a relatively high net clinical benefit when the high-risk threshold exceeded 0.2 (Fig 4C). To validate the robustness of these findings, an external independent cohort was introduced, where patients were classified into stable plaque and ruptured plaque groups. Given that plaque rupture typically represents a more severe and advanced pathological state, the stable vs. ruptured grouping was used to further evaluate the association between IGHG1 expression and plaque progression. The results showed that IGHG1 expression was significantly higher in the ruptured group compared to the stable group (Fig 4D). Moreover, ROC analysis confirmed that IGHG1 maintained excellent diagnostic performance in distinguishing stable from ruptured plaques in the external cohort (AUC = 0.911, Fig 4E), and DCA indicated favorable net clinical benefit (Fig 4F).

### 3.4. scRNA-seq analysis shows that IGHG1 signal was mainly detected in the NK-cell cluster in this dataset

**Fig 5:**
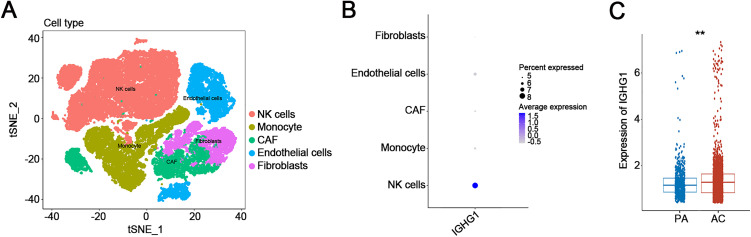
IGHG1 signal was mainly detected in the SingleR/CellMarker-annotated NK-cell cluster in this dataset. (A). Five cell types are annotated. (B). Expression of IGHG1 in different cell types. (C). Expression of IGHG1 between PA and AC groups in NK cells; **p < 0.01.

To further investigate the potential association between IGHG1 and carotid plaque progression at the cellular level, we conducted an in-depth analysis based on scRNA-seq data. This study included the AC and matched PA from three patients with calcified AS. The gene features, gene count, and mitochondrial gene percent in cells were shown in S2A Fig. After quality control, a total of 46,081 high-quality cells were retained for downstream analysis (S2B Fig). To minimize noise, 11 statistically significant principal components (p < 0.05) were selected based on the JackStraw analysis for dimensionality reduction (S2C Fig). Using the t-SNE algorithm, we clustered the cells into 26 distinct clusters (S2D Fig). Cell-type annotation was performed using an automatic matching strategy based on SingleR and the CellMarker database. Based on the best-matched reference annotations, these 26 clusters were annotated into five major cell types: NK cells, monocytes, cancer-associated fibroblasts (CAFs), endothelial cells, and fibroblasts (Fig 5A). We then assessed IGHG1 signal across the five automatically annotated cell groups. In this dataset, IGHG1 signal was mainly detected in the NK-cell cluster (Fig 5B). Moreover, the expression level of IGHG1 in AC tissues was significantly higher than in PA tissues in NK cells (p < 0.01, Fig 5C).

### 3.5. Cell–cell communication and pseudotime analysis of NK cell subtypes in carotid plaques

**Fig 6:**
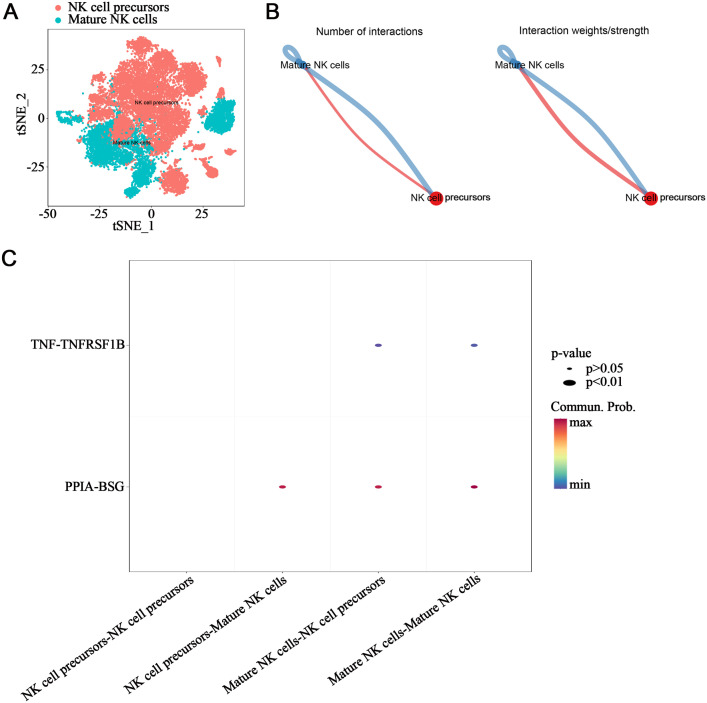
Cell-cell communication between NK cell subpopulations. (A). Two subpopulations of NK cells are annotated. (B). Cell-cell communication between mature NK cells and NK cell precursors. (C). Ligand–receptor pairs between mature NK cells and NK cell precursors.

Based on the previous analysis, we further refined the annotation of NK cell subpopulations and identified two distinct subtypes: NK precursor cells and mature NK cells (Fig 6A). To explore potential intercellular communication between these two NK subtypes, we performed ligand–receptor interaction analysis (Fig 6B–C). The results revealed significant intercellular interactions between NK precursor cells and mature NK cells (Fig 6B). PPIA–BSG and TNF–TNFRSF1B were identified as prominent predicted ligand–receptor pairs between the two annotated NK-cell-related subclusters, with PPIA–BSG showing a particularly strong inferred interaction signal (Fig 6C).

**Fig 7:**
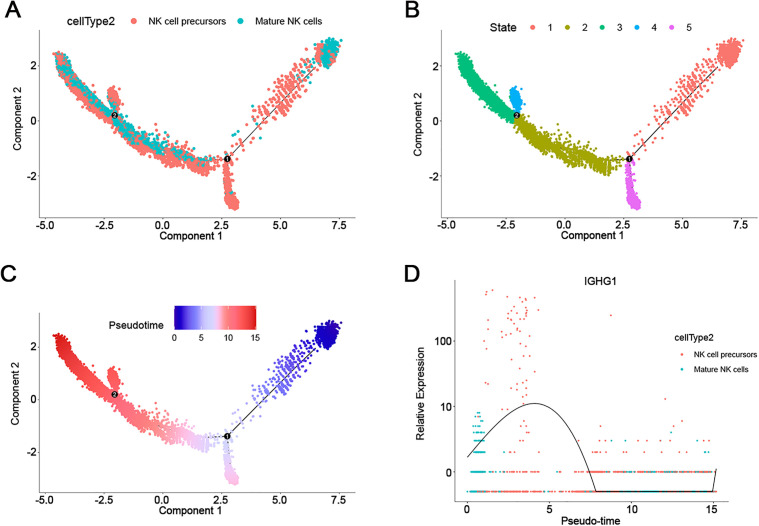
Pseudotime analysis of NK cells in carotid plaque. (A). Cell differentiation trajectory of NK cells. (B). Cell differentiation states of NK cells. (C). Pseudotime value of NK cells. (D). Expression dynamics of IGHG1 along the pseudotime axis.

To further dissect the differentiation trajectory of NK cell subtypes, we conducted pseudotime analysis using Monocle. The results showed that NK precursor cells and mature NK cells followed distinct differentiation trajectories (Fig 7A). Cell state analysis classified the cells into five states; NK precursor cells were predominantly enriched in States 1–5, whereas mature NK cells were mainly clustered in States 1, 2 and 3 (Fig 7B). The distribution of pseudotime values confirmed this trend (Fig 7C): as pseudotime progressed, NK cells gradually transitioned from a precursor to a mature state. Gene expression dynamics revealed that IGHG1 expression exhibited a transient peak followed by a sharp decline during differentiation (Fig 7D), suggesting that IGHG1 may represent a trajectory-associated signal during the inferred transition between the annotated NK-cell-related states, rather than direct evidence of a regulatory role in NK-cell maturation.

### 3.6. GSEA reveals immune pathways associated with IGHG1 expression

**Fig 8:**
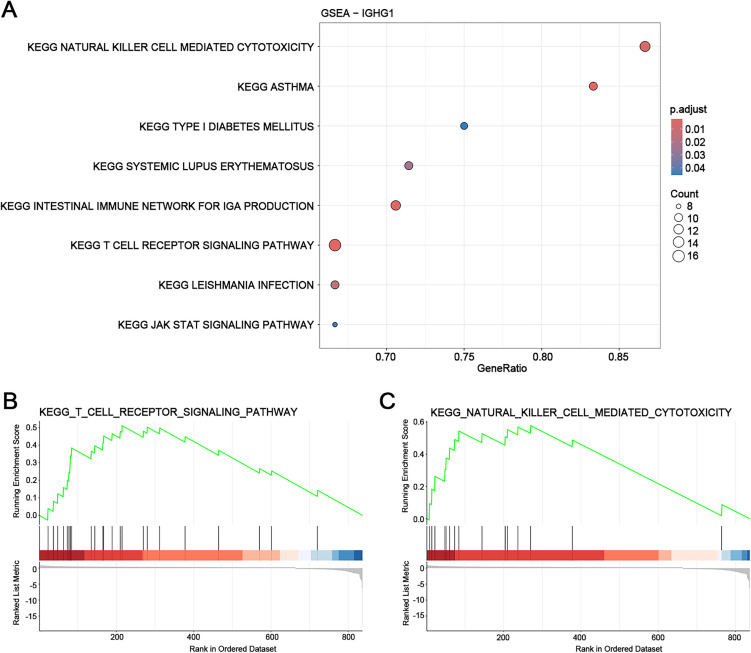
GSEA identifies immune-related pathways associated with IGHG1 expression. (A) GSEA based on KEGG pathways revealed eight significantly enriched pathways. B–C. Enrichment plots showing that high IGHG1 expression was positively associated with the activation of (B) the T cell receptor signaling pathway and (C) NK cell-mediated cytotoxicity.

To further investigate the potential functions and biological mechanisms of IGHG1 in carotid plaque progression, we performed GSEA based on KEGG pathways. The results identified eight significantly enriched pathways (Fig 8A). Among them, the top two pathways were the T cell receptor signaling pathway and NK cell-mediated cytotoxicity. These two pathways were closely related to immune regulation. We subsequently focused on analyzing the relationship between IGHG1 expression and these two immune-related pathways. As shown in Fig 8B–C, elevated IGHG1 expression was positively associated with the activation of the T cell receptor (TCR) signaling pathway and NK cell-mediated cytotoxicity. These findings indicated that IGHG1-correlated genes were associated with immune-related transcriptional programs in bulk transcriptomic data.

## 4. Discussion

In this study, we integrated bulk RNA-seq and scRNA-seq data to explore immune features associated with carotid plaque progression. Our analyses identified IGHG1 as a candidate gene associated with advanced plaque stage in the analyzed public datasets and further linked this signal to NK cell-related immune features within the available analytical framework. Rather than providing definitive mechanistic evidence, the present work offers a hypothesis-generating multi-omics perspective on immune-associated transcriptional changes during carotid plaque progression.

In the initial analysis, the 43 plaque progression-related DEGs were mainly enriched in immune-related biological processes and pathways, supporting immune remodeling as an important feature of carotid plaque progression [21–23]. As key components of the immune microenvironment, immune cells have long been recognized for their pivotal roles in AS plaque development. Most previous studies have focused on macrophages, T cells, and monocytes in the formation and progression of plaques [10,18,24,25]. However, some evidence suggests that innate immune responses may also be associated with larger AS plaque volumes [26]. Zheng et al. reported significant NK cell infiltration in carotid plaques [27], and Bonaccorsi et al. further revealed differential NK cell infiltration between early and advanced plaques [13]. Consistent with these findings, our results demonstrate significantly higher NK cell infiltration in advanced carotid plaques, suggesting that NK cells may play a critical role in promoting inflammation and regulating immune responses during plaque progression.

Furthermore, our results also revealed a dynamic transition of NK cells from a precursor to a mature state as the disease advanced, with mature NK cells enriched in advanced plaques. This finding is consistent with previous literature, which has shown that NK cells can promote lesion progression in AS driven by chronic pathogenic infections by sustaining a pro-inflammatory environment [28]. In addition, systemic overactivation of NK cells is considered a key mechanism contributing to the exacerbation of AS [29]. NK cells can promote CD4 ⁺ T cell differentiation into the Th1 subset by secreting IFN-γ early in lymph nodes, thereby amplifying the inflammatory response. Simultaneously, their expression of various chemokine receptors confers high migratory capacity, enabling effective recruitment to arterial lesions to exert immune functions [30]. Notably, our cell–cell communication analysis revealed a significant PPIA (CypA)–BSG (CD147) ligand–receptor interaction within the NK cell lineage. Previous studies have confirmed that the CypA–CD147 signaling pathway can induce MMPs, enhance local inflammation, and promote extracellular matrix degradation [31]. Moreover, inhibitors targeting the CypA–CD147 axis have been shown to slow plaque progression and stabilize vulnerable plaques in AS mouse models [32]. Therefore, we speculate that the PPIA–BSG signaling axis may not only regulate NK cell maturation and functional remodeling but also promote plaque instability by modulating matrix degradation and immune inflammation.

We further explored molecular markers associated with NK cells. Among all candidate genes, IGHG1 was prioritized due to its high predictive performance and strong association with immune-related pathways. IGHG1 encodes the constant region of the IgG1 heavy chain and is traditionally considered a plasma cell–specific product, known to be involved in the progression of various cancers [33–35]. Recent studies have linked IgG N-glycosylation to carotid plaque events [36,37]. Impaired IgG-Fc functionality has been shown to suppress NK cell cytotoxicity [38]. To date, research on IGHG1 in the context of carotid plaques and NK cells remains limited. One prior study reported increased IGHG1 expression in late-stage AS plaques compared to early-stage lesions [39]. In our study, both logistic regression and SHAP analysis indicated that IGHG1 had superior predictive power for plaque staging compared to other candidates, supporting its potential as a novel immune biomarker. Validation analyses showed that IGHG1 demonstrated high diagnostic accuracy and AUC values in both the training (0.831) and external validation cohorts (0.911), with consistent performance in distinguishing early vs. advanced and stable vs. ruptured plaques. These findings highlight its translational potential as a biomarker for high-risk plaques. In recent years, increasing attention has been given to early risk assessment and molecular subtyping of AS plaques through immune markers [40–42]. Our findings align with this trend and provide IGHG1 as a potential and new molecular tool in this context.

The association between IGHG1 expression and estimated NK-cell infiltration, together with the scRNA-seq and GSEA results, suggests that IGHG1 may be linked to NK cell-related immune features in carotid plaques. A recent study also reported that NK cell–related genes in AS were prominently enriched in this same pathway [43]. Additionally, our results showed that high IGHG1 expression was associated with the activation of the TCR signaling pathway, which plays a pivotal role in immune cell recruitment, co-activation, and amplification of inflammation [44–46]. The TCR pathway not only drives T cell activation and polarization but also enhances NK cell survival and effector function through cytokines such as IL-2 and IL-15 [47]. Previous research has highlighted the critical role of TCR signaling in shaping the immune microenvironment of atherosclerotic plaques [47]. IGHG1 may function as an upstream regulator in this intercellular communication network, enhancing NK cell responses and contributing to a positive feedback loop of inflammation within the plaque microenvironment.

Despite the multi-omics integrative approach used in this study, several limitations remain. First, the transcriptomic data were obtained from public databases, limiting control over clinical variables and potentially introducing bias. Second, the function of IGHG1 in NK cells has not yet been experimentally validated, and further mechanistic investigations are required. Third, the analytical workflow involved multiple computational methods, including WGCNA, LASSO, logistic regression, SHAP, CIBERSORT, CellChat, and pseudotime analysis; given the limited sample size and dataset heterogeneity, potential overfitting and reduced robustness cannot be excluded. Fourth, IGHG1 is a canonical B/plasma-cell-associated immunoglobulin gene. The IGHG1 signal observed in the annotated NK-cell cluster may be influenced by reference-dependent annotation, unresolved B/plasma-cell populations, doublets, or ambient RNA contamination. Finally, no experimental validation, such as protein-level confirmation, flow cytometry, spatial transcriptomics, or functional assays, was performed; therefore, the biological role of IGHG1 in carotid plaque progression remains to be validated.

## 5. Conclusion

Through integrative analysis of public bulk transcriptomic and single-cell datasets, this study identified IGHG1 as a candidate gene associated with carotid plaque progression and NK cell-related immune features. In the analyzed scRNA-seq dataset, IGHG1 signal was mainly detected in the automatically annotated NK-cell cluster; however, given the canonical association of IGHG1 with B-lineage and plasma cells, this finding should be interpreted cautiously. Overall, IGHG1 may represent an immune-associated transcriptional signal linked to plaque progression, but its cellular origin and functional relevance require further experimental validation.

## Supporting information

S1 Fig CIBERSORT-based estimation of immune cell infiltration in early-stage and advanced-stage carotid plaques. (TIF)

S2 Fig scRNA-seq analysis. A-B. Quality control of the scRNA-seq data. C. JackStraw plot revealed the significant PCs. D. A total of 26 cell clusters were identified.
(TIF)

S1 Table Differentially expressed genes associated with carotid plaque stages. (XLS)
