# Supplementary figures and images for: Association of IGHG1 with carotid plaque progression and NK Cell-related immune features: Insights from bulk and single-cell transcriptomics
Source: PLoS One. 2026 Aug 3;21(8):e0353523. doi: 10.1371/journal.pone.0353523 (PMC13432142; doi:10.1371/journal.pone.0353523)

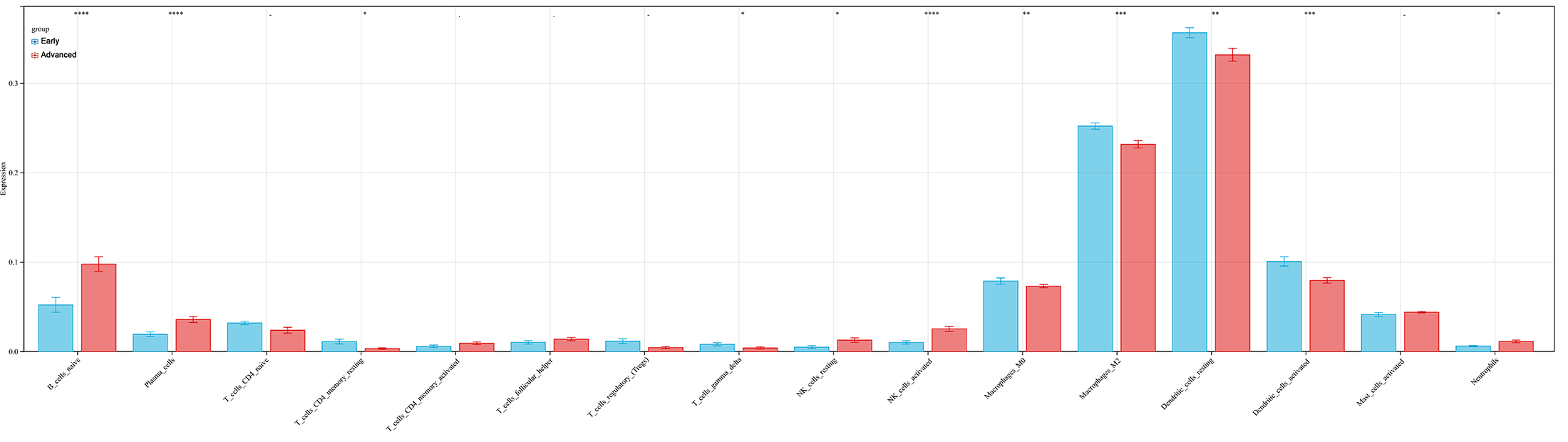

Supplement: S1 Fig — (TIF) [file pone.0353523.s001.tif]

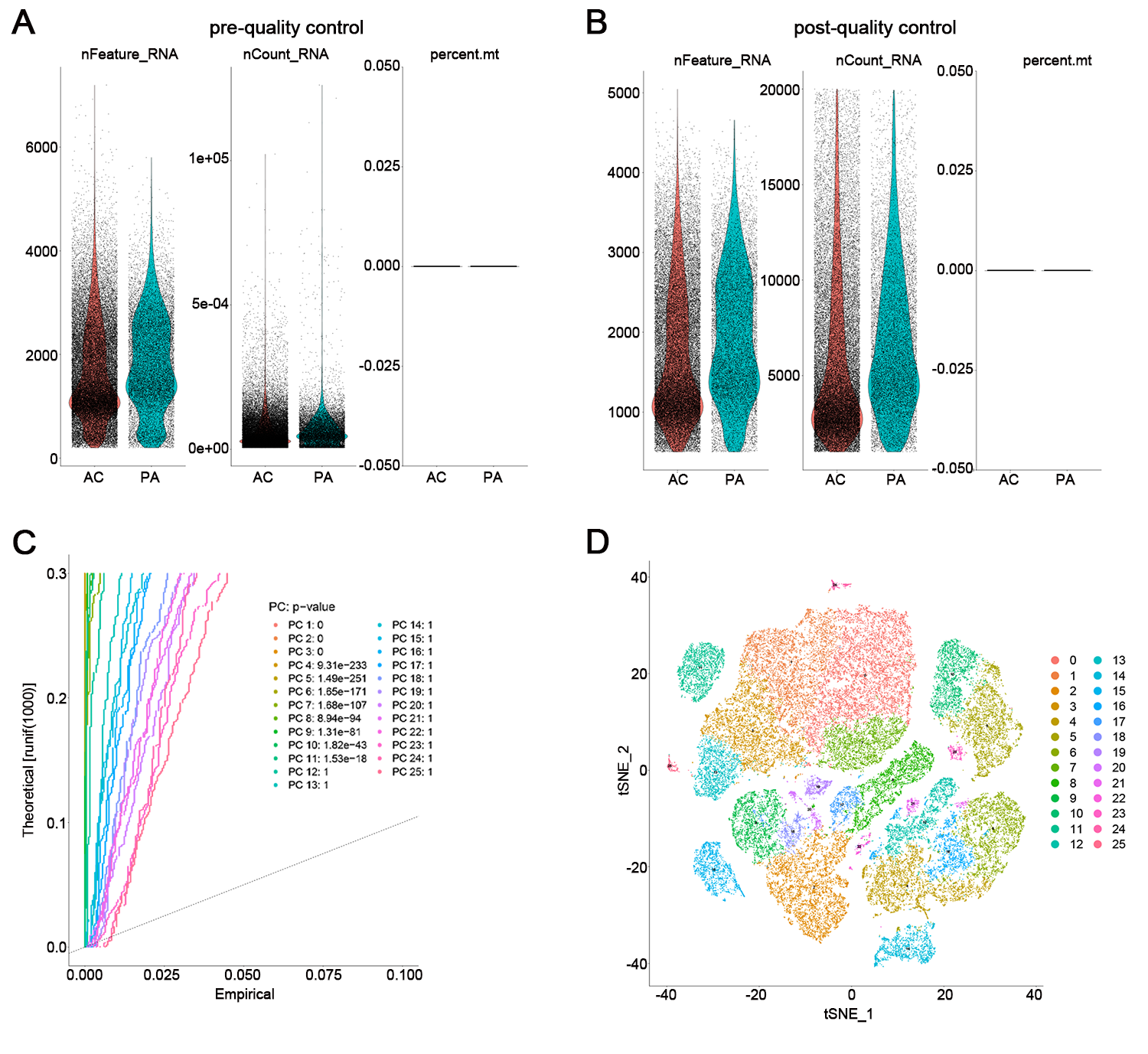

Supplement: S2 Fig — A-B. Quality control of the scRNA-seq data. C. JackStraw plot revealed the significant PCs. D. A total of 26 cell clusters were identified. (TIF) [file pone.0353523.s002.tif]
